# Development of a plasmid-based expression system in *Clostridium thermocellum* and its use to screen heterologous expression of bifunctional alcohol dehydrogenases (*adhE*s)

**DOI:** 10.1016/j.meteno.2016.04.001

**Published:** 2016-04-22

**Authors:** Shuen Hon, Anthony A. Lanahan, Liang Tian, Richard J. Giannone, Robert L. Hettich, Daniel G. Olson, Lee R. Lynd

**Affiliations:** a Thayer School of Engineering, Dartmouth College, Hanover, NH, USA; b BioEnergy Science Center, Oak Ridge, TN, USA; c Chemical Sciences Division, Oak Ridge National Laboratory, Oak Ridge, TN, USA

**Keywords:** *Clostridium Thermocellum*, Plasmid, *adhE*, Structural stability, Gene expression

## Abstract

*Clostridium thermocellum* is a promising candidate for ethanol production from cellulosic biomass, but requires metabolic engineering to improve ethanol yield. A key gene in the ethanol production pathway is the bifunctional aldehyde and alcohol dehydrogenase, *adhE*. To explore the effects of overexpressing wild-type, mutant, and exogenous *adhE*s, we developed a new expression plasmid, pDGO144, that exhibited improved transformation efficiency and better gene expression than its predecessor, pDGO-66. This new expression plasmid will allow for many other metabolic engineering and basic research efforts in *C. thermocellum*. As proof of concept, we used this plasmid to express 12 different *adhE* genes (both wild type and mutant) from several organisms. Ethanol production varied between clones immediately after transformation, but tended to converge to a single value after several rounds of serial transfer. The previously described mutant *C. thermocellum* D494G *adhE* gave the best ethanol production, which is consistent with previously published results.

## Highlights

• Developing of robust and reliable *C. thermocellum* expression plasmid.
• Analysis on causes of plasmid instability and poor transformation efficiency.
• Comparison of ethanol production from different *adhE*s.

## 1 Introduction

*Clostridium thermocellum* is a good candidate for producing biofuels from cellulosic biomass via consolidated bioprocessing (Olson et al., 2012). This microorganism is among the most effective described at solubilizing lignocellulose (Lynd et al., 2002), and ferments glucose and glucan oligomers to organic acids, hydrogen, and ethanol. In recent years, there have been attempts (Argyros et al., 2011, Biswas et al., 2015, Biswas et al., 2014, Deng et al., 2013, Papanek et al., 2015) at engineering *C. thermocellum* to produce ethanol as the sole product at high yield; these attempts thus far have fallen short of the high yields achieved by conventional ethanol producers such as yeast and *Zymomonas*.

Of the existing and reported genetic engineering efforts in *C. thermocellum*, most have taken the approach of gene deletions (Argyros et al., 2011, Biswas et al., 2015, Olson et al., 2010, Papanek et al., 2015, Rydzak et al., 2015, Tripathi et al., 2010, van der Veen et al., 2013). There have been a few reports of gene expression, or over expression, in *C. thermocellum* (Deng et al., 2013, Lo et al., 2015, Olson et al., 2013, Zheng et al., 2015), but methodologies are in general less well developed than for gene deletion. One example related to metabolic engineering is the expression of the *Thermoanaerobacterium saccharolyticum* pyruvate kinase in *C. thermocellum* (Deng et al., 2013). Another example is the complementing of *adhE* activity in *C. thermocellum adhE* deletion strain (Lo et al., 2015, Zheng et al., 2015). In these cases, gene expression was achieved via targeted recombination of the gene of interest onto the chromosome, a process that takes several weeks under ideal conditions (Olson and Lynd, 2012a).

Plasmid-based gene expression, on the other hand, can be performed in a single step, and therefore lends itself to higher throughput metabolic engineering applications and thus is especially relevant during screening processes. Related prior work includes an attempt to complement the *cipA* deletion in *C. thermocellum*, and resulted in partial (~33% of wild type) restoration of Avicel solubilization (Olson et al., 2013). Efforts to identify native *C. thermocellum* promoters for use in expressing genes encountered issues with obtaining consistent and reliable results with reporter enzyme activities (Olson et al., 2015).

Here, we report improvements to a *C. thermocellum* expression plasmid, and use this improved plasmid to screen a variety of different *adhE*s for improved ethanol production in the *C. thermocellum adhE* deletion strain, LL1111.

## 2 Materials and methods

### 2.1 Plasmid and strain construction

Table 1 lists the strains and plasmids used or generated in this study; Table S1 lists the primers used in this study. Plasmids were constructed via the isothermal assembly method (Gibson, 2011), using a commercial kit sold by New England Biolabs (Gibson Assembly^®^ Master Mix, product catalog number E2611). DNA purification was performed using commercially available kits from Qiagen (Qiagen catalog number 27,106) or Zymo Research (Zymo Research catalog numbers D4002 and D4006). Transformation of *C. thermocellum* was performed using previously described methods (Olson and Lynd, 2012a); all plasmid DNA intended for transforming into *C. thermocellum* was propagated and purified from *Escherichia coli* BL21 derivative strains (New England Biolabs catalog number C2566) to ensure proper methylation of plasmid DNA (Guss et al., 2012).

**Table 1:** List of strains and plasmids used in this study.

**Strains**	**Organism**	**Description**	**Accession number**	**Reference or source**
*E. coli* T7 express	*Escherichia coli*	*fhuA2 lacZ: :T7 gene1 [lon] ompT gal sulA11 R(mcr-73: :miniTn10–*Tet^S^*)2 [dcm] R(zgb-210: :Tn10–*Tet^S^*) endA1 Δ(mcrC-mrr)114: :IS10*		New England Biolabs
LL1004	*C. thermocellum*	DSM 1313	CP002416	DSMZ
LL1111	*C. thermocellum*	DSM1313 ∆*hpt* ∆*adhE ldh*(R175L)	SRX744221	Lo et al. (2015)
LL1153	*C. thermocellum*	Strain LL1111 with two forms of plasmid pSH007; the full length version, and a truncated version where *adhE* is deleted		This study
LL1154	*C. thermocellum*	Serial transfer of strain LL1153; plasmid pSH007 spontaneously integrated into the *gapdh* promoter region via homologous recombination		This study
LL1160	*C. thermocellum*	LL1111 *adhE*^*+*^*ldh*(R175L)	SRA273168	Lo et al., 2015, Zheng et al., 2015
LL1161	*C. thermocellum*	LL1111 *adhE*^*+*^D494G *ldh*(R175L)	SRA273169	Zheng et al. (2015)
adhE*	*C. thermocellum*	Ethanol tolerant strain of *C. thermocellum*		Brown et al. (2011)
LL1231	*C. thermocellum*	DSM 1313 Δ*hpt* Δ*ldh* Δ*pta*-*ack* Δ*hydG* Δ*pfl adhE*(D494G P525L)		This study
LL1025	*Thermoanaerobacterium saccharolyticum*	Strain JW/YS-485L	CP003184	Shaw et al. (2008a)
LL1040	*T. saccharolyticum*	Ethanologen *T. saccharolyticum* strain ALK2; genotype ∆*ldh: :erm* ∆*(pta-ack): :kan*	SRA233066	Shaw et al. (2008b)
LL1049	*T. saccharolyticum*	Ethanologen *T. saccharolyticum* strain; genotype ∆*(pta-ack)* ∆*ldh* Δ*or795: :metE-ure* Δ*eps*. This strain is also know n as strain M1442	SRA233073	Shaw et al. (2012)
LL1115	*Thermoanaerobacter ethanolicus*	Strain JW200		ATCC
LL1053	*Thermoanaerobacterium thermosaccharolyticum*	DSM 571		DSMZ
LL451	*Clostridium straminisolvens*	DSM 16,021		DSMZ
LL447	*Clostridium clariflavum*	DSM 19,732		DSMZ
LL1232	*Geobacillus thermoglucosidasius*	ATCC 43,742		ATCC
LL1258	*Thermoanaerobacter mathranii*	DSM11426		DSMZ
**Plasmids**				
pDGO-66		Expression vector		Olson et al. (2015)
pSH007		pDGO-66 with DSM1313 clo1313_1798 cloned in at *Pvu*II site		This study
pDGO125		Improved expression vector, lacking annotated SSO		This study
pDGO143		pDGO125 with insulator sequence between MCS and cat gene promoter		This study
pDGO126		Improved expression vector, contains annotated SSO		This study
pDGO144		pDGO126 with insulator sequence between MCS and cat gene promoter		This study
**adhE expression plasmids**		All plasmids used clo1313_2638 promoter to drive expression of the *adhE*; both promoter and gene were cloned into the *Hin*dIII site at the MCS in pDGO144		
pLL1119		*C. thermocellum* wild type *adhE* (clo1313_1798)		This study
pLL1120		*C. thermocellum adhE* D494G		This study
pLL1121		*C. thermocellum adhE* P704L H734R, also known as AdhE*		Brown et al. (2011)
pLL1122		*C. thermocellum adhE* D494G P525L		This study
pLL1123		*T. saccharolyticum* wild type *adhE* (Tsac_0416)		This study
pLL1124		*T. saccharolyticum adhE* V52A K451N; 13 aa repeat, also known as ALK2		Shaw et al. (2008b)
pLL1125		*T. saccharolyticum adhE* G544D		This study
pLL1126		*T. mathranii* wild type *adhE* (Tmath_2110)		This study
pLL1127		*G. thermoglucosidasius* wild type *adhE* (Geoth_RS19255)		This study
pLL1128		*T. thermosaccharolyticum* wild type *adhE*		This study
pLL1129		*C. clariflavum* wild type *adhE* (Clocl_0117)		This study
pLL1130		*T. ethanolicus* wild type *adhE* (Genbank DQ836061.1)		This study
pLL1131		*C. straminisolvens* wild type *adhE* (JCM21531_3461 to JCM21531_3464)		This study

### 2.2 Re-designing the expression plasmid

**Fig. 1:**
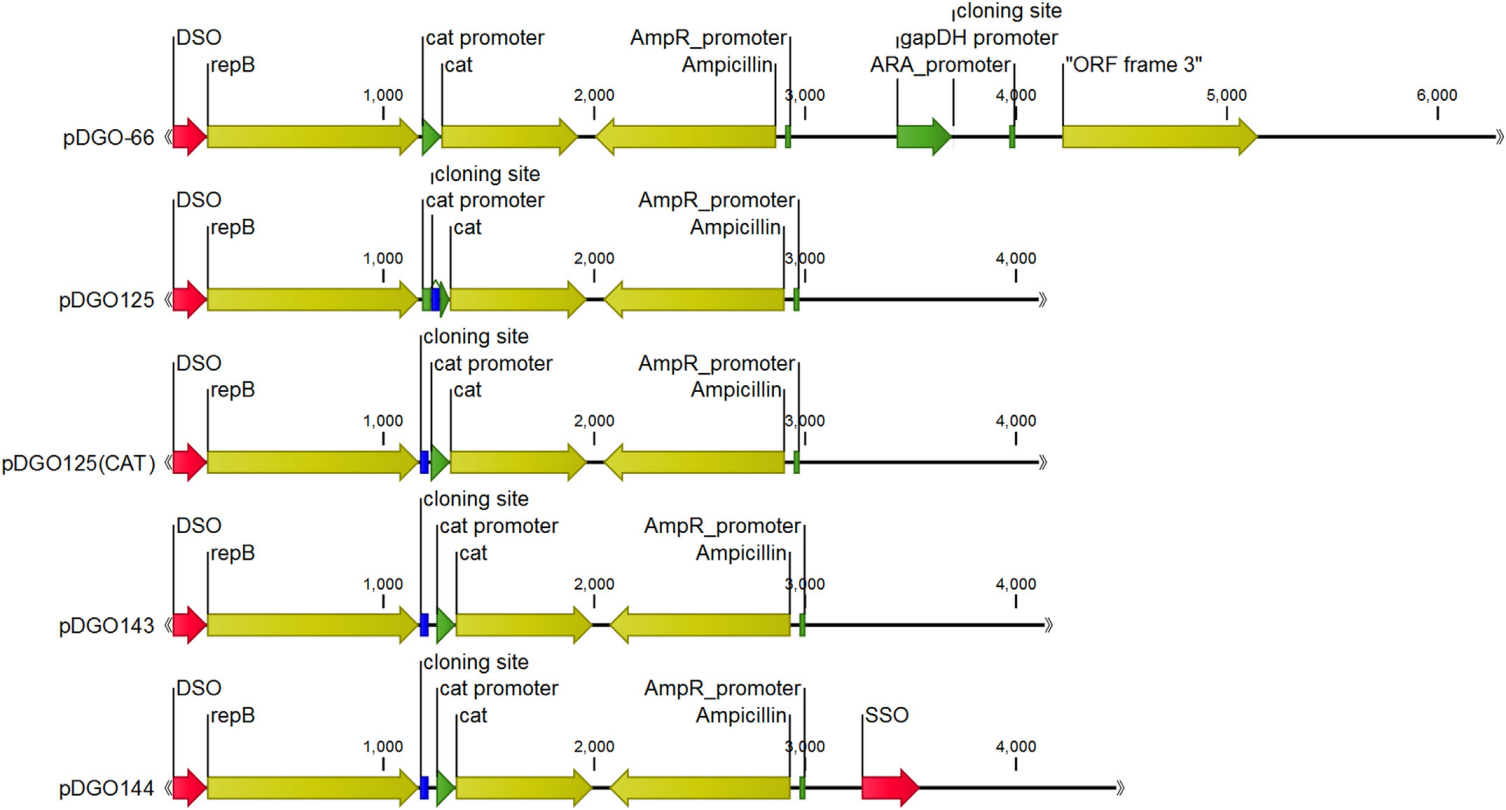
Functional organization of key plasmids. From top to bottom: pDGO-66 starting vector; pDGO125 relocating the cloning site from after *repB*-*cat* to between the two genes (resulting in *cat* promoter becoming disrupted); pDGO125(CAT) moving the cloning site from within the *cat* promoter to upstream; pDGO143 inserting an insulator sequence between the cloning site and the *cat* promoter; pDGO144 including a broad-host range SSO into the plasmid. The associated impacts on transformation efficiencies for the plasmids shown here are noted in Table 2.

Fig. 1 and S1 shows the features of the various expression plasmids and the intermediates. We first removed the *Pvu*II cloning site on our older expression plasmid, pDGO-66, in favor of a multiple cloning site (MCS), and inserted this MCS to the intergenic region between replication initiator gene *repB* and the thiamphenicol resistance gene, *cat* (Olson and Lynd, 2012b), thus placing the gene of interest between two genes that are essential for plasmid selection. We also eliminated the *gapDH* promoter from the plasmid to allow us the flexibility to use different promoters. The resulting plasmid was named pDGO125. A single-strand origin of replication (SSO) (Boe et al., 1989) was also added upstream of the double-strand origin of replication (DSO) in pDGO125, as there was no canonical SSO in plasmid pDGO-66; the resulting plasmid was named pDGO126. We later identified a promoter region upstream of the *cat* gene that we had disrupted with the MCS in plasmids pDGO125 and pDGO126; we thus moved the MCS to be upstream of the cat promoter region in both plasmids to generate pDGO125cat and pDGO126cat. Lastly, a 27 bp “insulator” sequence was introduced into plasmids pDGO125cat and pDGO126cat between the MCS and the *cat* promoter region, resulting in plasmids pDGO143 and pDGO144, respectively. All *adhE* expression plasmids used the Clo1313_2638 promoter (Olson et al., 2015) to drive expression of the *adhE* gene. Both the promoter and gene were cloned into the HindIII site at the MCS in plasmid pDGO144.

### 2.3 Determining the segregational and structural stability of plasmids

Plasmids were transformed into *C. thermocellum* strain LL1004 (wild type), colonies were picked, and the presence of the plasmid was verified by PCR with primers XSH0210 and XSH0211. To determine plasmid structural stability after transformation into *C. thermocellum*, plasmid DNA was isolated from transformants and analyzed by PCR and restriction digestion. To determine segregational stability, cultures of *C. thermocellum* strain LL1004 bearing the respective plasmids were grown with or without thiamphenicol selection, and the fraction of plasmid-containing colonies was determined by dilution plating, with and without thiamphenicol selection. Plasmid DNA from *C. thermocellum* was prepared using the Qiagen DNA miniprep kit, with the added step of incubating the harvested and re-suspended cells with Epicentre Ready-Lyse™ lysozyme solution (Epicentre catalog number R1804m) at 37 °C for 30 min in buffer P1, before proceeding with the rest of the miniprep protocol, following the instructions of the manufacturer.

### 2.4 Media and growth conditions

All chemicals were of molecular grade, and were obtained from either Sigma Aldrich or Fisher Scientific, unless otherwise specified. *C. thermocellum* strains were grown in anaerobic chambers (Coy Laboratory Products, Grass Lakes, MI, USA) at 55 °C, with the hydrogen concentrations in the chamber maintained at greater than 1.5%. Two media formulations were used, with both containing 5 g/L cellobiose (Sigma C7252) as the primary carbon source: complex medium CTFÜD (Olson and Lynd, 2012a) with initial pH of 7.0 (pH measured at room temperature) was used for growing competent *C. thermocellum* cells for transformation, as well as for recovery post-electroporation and initial plasmid tests. Defined medium MTC (Ozkan et al., 2001, Zhang and Lynd, 2003) with initial pH of 7.4 at room temperature was used to determine ethanol production from the various *adhE*s. Where needed, thiamphenicol dissolved in dimethyl sulfoxide (DMSO) was added to the cultures to a final concentration of 6 µg/ml. When switching strains from CTFÜD medium to MTC medium, the strains were transferred 3 times at a 1:100 dilution each time to remove any yeast extract carried over from the CTFÜD medium.

### 2.5 Biochemical assays

Cultures for the ethanol and cellobiose assays were inoculated with 2% inoculum, and then grown anaerobically at 55 °C for 72 h. Cells were pelleted by centrifugation (5 min at >20,000 *g*), and the supernatant was used in the assays. The concentration of ethanol in the cultures was determined via ADH enzyme assay in the acetaldehyde and NADH-producing direction (Bisswanger, 2011). The reaction had the following component concentrations: 67 mM sodium pyrophosphate, 20 mM glycine, 1 mM semicarbazide, 8.3 mM NAD^+^, and 0.1 U/ml alcohol dehydrogenase enzyme (Sigma A3263); 20 µL of sample was used in a 200 µL reaction volume. The reactions were followed on a microplate reader by monitoring the increase in absorbance at 340 nm (i.e. NADH accumulation) and comparing the results against known standards.

Cellobiose assays were adapted from glucose determination assays (Bisswanger, 2011) in that a beta-glucosidase (Novozymes 188, formerly sold by Sigma as product C6105) was included in the reaction mixture. The reaction was followed on a microplate reader by monitoring the increase in absorbance at 340 nm (i.e. NADPH accumulation). Reaction rates were determined from a linear region of the absorbance curve; standard curves were generated using solutions with known cellobiose concentrations.

### 2.6 Measuring *adhE* expression

*adhE* expression was measured via reverse transcription quantitative PCR (RT-qPCR). Strains were cultured in 5 ml MTC-5 defined medium, and harvested in log-phase (OD600 0.6–0.8); 0.6 ml aliquots of the cell cultures were immediately treated with RNA protect Bacteria Reagent (Qiagen catalog number 76,506) and stored at −80 °C until time for RNA purification. RNA purification, cDNA synthesis, and qPCR were performed as previously described (Zhou et al., 2015); the primers used for qPCR are described in Table S1. *adhE* expression in each strain was normalized against *recA* expression (Livak and Schmittgen, 2001) to allow for comparison of *adhE* expression across the strains.

### 2.7 Sequencing

Routine Sanger sequencing was performed by Genewiz Inc.; whole genome resequencing of strains was performed by the Department of Energy Joint Genome Institute. Sequence data was analyzed with the CLC Genomics Workbench version 7 (Qiagen Inc.). Sequencing data is available for strains LL1153 and LL1154 from the Sequence Read Archive; the accession numbers are SRA278181 and SRA278180.

### 2.8 Proteomic analyses

The abundance of AdhE protein expressed in each strain was measured by liquid chromatography tandem mass spectrometry (LC-MS/MS) in technical duplicate. For each measurement, 45 ml of culture grown in MTC defined medium was used. Cells were harvested in mid-log phase (OD600=0.5–0.8). The fermentation products from an aliquot of the same culture were measured by high pressure liquid chromatography (HPLC) as previously described (Holwerda et al., 2014). Cells were pelleted, washed, and processed for LC-MS/MS-based proteomic analysis as previously described (Giannone et al., 2011). Briefly, cell pellets were resuspended in sodium dodecyl sulfate lysis buffer, boiled for 5 min and pulse-sonicated. Two milligrams of the resulting whole-cell protein extract was precipitated by trichloroacetic acid, pelleted, washed and air-dried. The pelleted protein was then resuspended in urea–dithiothreitol, cysteines blocked by iodoacetamide and proteins digested to peptides via two 20 μg additions of sequencing-grade trypsin (Sigma Aldrich). Proteolyzed samples were then salted, acidified and filtered through a 10 kDa MWCO membrane (Vivaspin 2; GE Healthcare).

Peptides from each sample were quantified by BCA assay (Pierce) and 5 µg analyzed via nanospray LC-MS/MS using a LTQ-Orbitrap XL mass spectrometer (Thermo Scientific) operating in data-dependent acquisition (one full scan at 15k resolution followed by 10 MS/MS scans in the LTQ, all one µscan). Each 5 µg peptide sample was separated by HPLC over a 120 min organic gradient. Resultant peptide fragmentation spectra (MS/MS) were searched against the *C. thermocellum* DSM 1313 proteome database concatenated with various AdhE proteins (Table S1), common contaminants, and reversed sequences to control false-discovery rates using Myrimatch v.2.1 (Tabb et al., 2008). Peptide spectrum matches were filtered by IDPicker v.3 (Ma et al., 2009) and assigned matched-ion intensities (MIT) based on observed peptide fragment peaks (Giannone et al., 2015). PSM MITs were summed on a per-peptide basis and only those uniquely and specifically matching a particular protein were moved onto subsequent analysis with InfernoRDN (Taverner et al., 2012). Peptide intensity distributions were log_2_-transformed, normalized by LOESS, and standardized by median centering across samples as suggested by InfernoRDN.

Before determining protein abundance, low quality peptides were removed based on the following criteria: Peptides not present in both technical replicates were removed. Peptides not present in all members of a strain group were removed. The *C. thermocellum* strain group included LL1004 and LL1111 with plasmids pLL1119, pLL1120, pLL1121 and pLL1122. Since LL1111 with plasmid pLL1119 does not have a full-length AdhE protein (being the AdhE deletion negative control), peptides that were only absent from that strain were not eliminated. Furthermore, there are a number of peptides that are unique to a specific mutation. For example, the peptides TFFDVS**P**DPSLASAK and TFFDVS**L**DPSLASAK differ by a single amino acid residue resulting from the P525L mutation in the AdhE protein from plasmid pLL1122. Peptides AYENGASD**P**VAR and AYENGASD**L**VAR differ by a single amino acid residue resulting from the P704L mutation in the AdhE protein from plasmid pLL1121. Similar examples are found in the *T. saccharolyticum* AdhE mutants. Since the appropriate variant of each peptide was found in its respective strain, so these peptides, and ones displaying similar patterns were not removed. The *T. saccharolyticum* group included strain LL1111 with plasmids pLL1123, pLL1124 and pLL1125. Other plasmids were not grouped.

For reference, the same analysis was performed for GapDH and Pfk, two proteins that play a key role in glycolysis and are often used as reference genes in quantitative PCR experiments (Supplemental Fig. S2A and S2B).

## 3 Results and discussion

### 3.1 Plasmid stability problems with pDGO-66

**Fig. 2:**
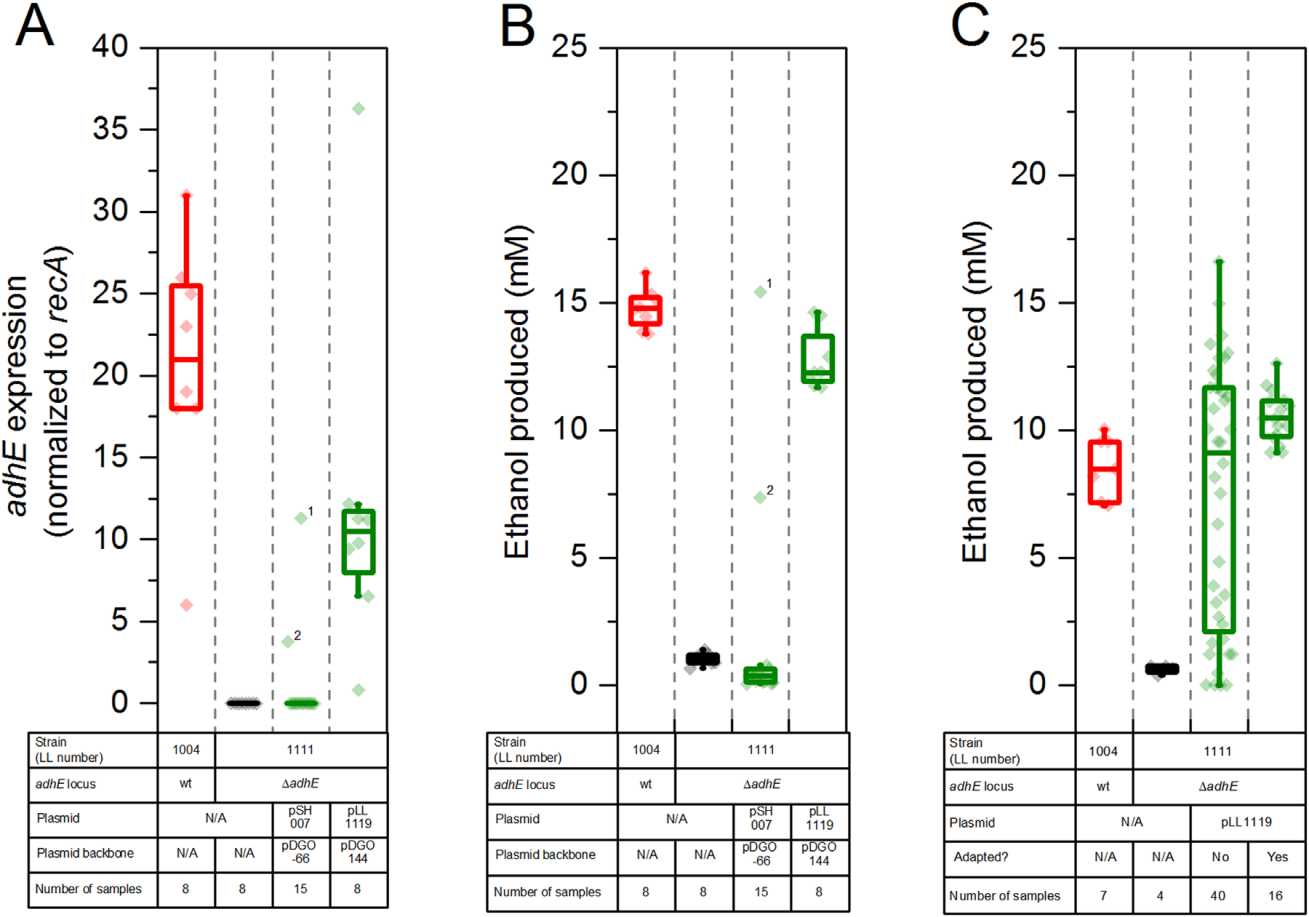
**A.***adhE* expression (normalized to *recA* expression) in wild type *C. thermocellum*, *adhE* deletion strain LL1111, and LL1111 complemented with pSH007 (older expression plasmid) or pLL1119 (newer expression plasmid) **B-C.** Ethanol production from wild type *C. thermocellum* (strain LL1004), *C. thermocellum adhE* deletion strain LL1111, and various methods of complementation. (B) shows the improvement in ethanol production obtained by switching from the pDGO-66 backbone to the pDGO144 backbone. This data was collected on MTC-5 defined medium with 6 µg/ml thiamphenicol. (C) shows the effect of serial transfer on ethanol production in rich medium (CTFÜD with 6 µg/ml thiamphenicol). Plasmid pLL1119 expresses the *C. thermocellum adhE* under control of the Clo1313_2638 promoter on the pDGO144 plasmid backbone. The box plot shows the 25–75th percentile range. Whiskers on the box plot represent 1.5× the interquartile range. Superscripts on data points in (A) and (B) represent data points for specific strains, ^1^LL1153 and ^2^LL1154, respectively.

In our first attempts to express *adhE* using plasmid pDGO-66, most colonies showed non-existent or low levels of ethanol production, although, initially, one colony gave high ethanol production (Fig. 2). One low-ethanol producing colony was named LL1153. Re-sequencing analysis of strain LL1153 revealed the presence of two forms of the plasmid pSH007: the full length version, and a version where the *adhE* gene had been deleted (Fig. S3). The full-length version represented only about 10% of all of the plasmid population, and may explain the low ethanol production of this strain, despite the maintenance of the plasmid antibiotic resistance phenotype.

Serial transfer of strain LL1153 resulted in an increase in ethanol production. We named this adapted strain LL1154 (this strain is shown in Fig. 2(B) as the data point exhibiting high ethanol production from the pDGO-66 based plasmid). Re-sequencing analysis of this strain revealed that the plasmid – and the *adhE* gene – had integrated into the genome at the *gapDH* locus, possibly by homologous recombination with the plasmid-based *gapDH* promoter region (see pDGO-66 plasmid map in Fig. S1). While we have long suspected that our plasmids were spontaneously integrating on the chromosome, here we provide direct evidence to support our hypothesis (Fig. S4). A recent report describing isobutanol production in *C. thermocellum* also documented the spontaneous integration of plasmid DNA onto the chromosome (Lin et al., 2015).

### 3.2 Improving plasmid structural stability

Based on our experience with plasmid pDGO-66, we determined that the low ethanol production was due to problems with structural stability, particularly loss of the *adhE* gene (Fig. S3). Plasmids that replicate via the rolling-circle method require both a double-strand origin of replication (DSO) and a single-strand origin of replication (SSO) (Khan, 2005). In plasmid pDGO-66, the DSO is upstream of the *repB* gene, but no SSO is known to exist in this plasmid. In some cases, plasmids without an SSO are still able to replicate, although the efficiency of replication is reduced, and the single-stranded DNA that accumulates can stimulate the formation of deletions (Bron et al., 1991) We inserted the broad-host-range SSO from plasmid pUB110 (Boe et al., 1989), which has an identical *repB* gene to that of plasmid pDGO-66. All of our initial plasmids were created both with and without the SSO. We looked at its effect on transformation efficiency, structural stability (Fig. S5) and segregational stability, and ultimately did not find any effect of its presence. One possibility is that this SSO is not recognized by *C. thermocellum*; another possibility is that the plasmid already contains a cryptic SSO.

Next, we moved the relative position of the gene expression cassette upstream of the antibiotic resistance marker. The purpose of this was to prevent the kind of truncation event observed with plasmid pSH007, since the plasmid would need both the replicon and the antibiotic resistance marker to function. Putting the multi-cloning site (MCS) upstream of the *cat* gene reduced transformation efficiency to 0 (plasmids pDGO125 and pDGO126). We suspected there might have been a problem with the particular MCS that we used, so we used a different MCS from plasmid pMU102 (MCS102), which is known to have high transformation efficiency (plasmids pDGO125(102MCS) and pDGO126(102MCS)). This did not improve transformation efficiency, so we tried using only the 6 bp recognition sequence of the PvuII restriction enzyme or eliminating the MCS entirely (plasmids pDGO125(PvuII), pDGO126(PvuII), pDGO125(no MCS) and pDGO126(no MCS)). In both cases, transformation efficiency improved. This led us to consider the possibility that we were disrupting a promoter of the *cat* gene. To address this problem, we moved the MCS 54 bp further upstream (101 bp upstream of the *cat* gene start codon). Finally, we added a 27 bp sequence of random DNA to “insulate” the *cat* promoter from the effect of the MCS. This final set of plasmids, pDGO143 and pDGO144, had transformation efficiencies as high as the pMU102 positive control (Table 2); Fig. 1 highlights the most important steps in the development of pDGO-66 to pDGO143/144.

**Table 2:** Transformation efficiencies of the plasmids that were developed in this study. Ratios were determined from three independent transformations of these plasmids into *C. thermocellum* strain LL1004 (wild type), normalized to pMU102 positive control's transformation efficiency. For transformation efficiency measurements, n=3.

**Plasmid name**	**Normalized transformation efficiency (CFU/µg DNA)**		**Annotated SSO included?**	***repB*****-*****cat*****orientation**	**Distance between an upstream feature and*****cat*****gene ATG**	**Description**	**Source**
	**Count**	**Standard deviation**					
pMU102	1.00	0.00	N	*repB*-*cat*-MCS2	106	Positive control plasmid	Olson and Lynd, 2012a, Olson and Lynd, 2012b
pDGO-66	0.20	0.13	N	*repB*-*cat*-*Pvu*II	106	*C. thermocellum* expression plasmid based on pDGO-37 with addition of *gapDH* promoter and Clo1313_1881 terminator	Olson et al. (2015)
pDGO125	0.00	0.00	N	*repB*-MCS1-*cat*	47	MCS original location	This study
pDGO125(102MCS)	0.00	0.00	N	*repB*-MCS2-*cat*	47	pMU102 MCS, original location	This study
pDGO125(*Pvu*II)	0.00	0.00	N	*repB*-*Pvu*II-*cat*	47	*Pvu*II site, original location	This study
pDGO125(no MCS)	4.35	5.40	N	*repB*-*cat*	106	no MCS	This study
pDGO125(CAT)	1.07	1.32	N	*repB*-MCS2-*cat*	101	MCS moved upstream of *cat* promoter	This study
pDGO143	1.51	1.33	N	*repB*-MCS2-insulator-*cat*	128^a^	MCS moved and insulator added	This study
pDGO126	0.00	0.00	Y	*repB*-MCS1-*cat*	47	SSO, MCS original location	This study
pDGO126(102MCS)	0.00	0.00	Y	*repB*-MCS2-*cat*	47	SSO, pMU102 MCS, original location	This study
pDGO126(*Pvu*II)	0.00	0.00	Y	*repB*-*Pvu*II-*cat*	47	SSO, *Pvu*II site, original location	This study
pDGO126(no MCS)	1.62	0.66	Y	*repB*-*cat*	106	SSO, no MCS	This study
pDGO126(CAT)	1.67	1.63	Y	*repB*-MCS2-*cat*	101	SSO, MCS moved upstream of *cat* promoter	This study
pDGO144	1.83	0.90	Y	*repB*-MCS2-insulator-*cat*	128^a^	SSO, MCS moved and insulator added	This study

a The insulator sequence is not counted as a feature; in pDGO143 and pDGO144, the feature used for determining this number is the MCS.

### 3.3 AdhE expression with the new plasmid

We tested the new plasmid by using it to express *adhE* in the LL1111 *adhE* deletion strain (Lo et al., 2015), This strain was chosen because it shows low levels of ethanol production, and also had low levels of *adhE* expression (Fig. 2(A)). The *adhE* gene is a good test case, because the AdhE protein is one of the highest-expressed proteins in *C. thermocellum* (Rydzak et al., 2012), and presumably similar levels of *adhE* expression are required for matching wild type levels of ethanol production.

Initial attempts to express *adhE* in the pDGO-66 backbone were largely unsuccessful. Out of 15 colonies screened, only 1 showed ethanol production greater than zero (this strain was later renamed LL1153, and subsequently adapted to generate strain LL1154, see plasmid stability discussion). By contrast, *adhE* expression in the pDGO144 plasmid backbone showed ethanol production at almost wild type levels for 8 out of 8 colonies tested (note that this was after serial transfer) (Fig. 2(B)).

To confirm that the improvement in ethanol production was due to improved expression of *adhE*, we compared normalized *adhE* expression in strains of LL1111 complemented either with pDGO-66 or pDGO144, expressing *C. thermocellum adhE* (pSH007 and pLL1119, respectively). We found that overall, the improved expression plasmid, pDGO144, more reliably resulted in high levels of *adhE* expression (i.e. comparable to expression levels in wild type *C. thermocellum*), whereas with pDGO-66, we saw in most cases that *adhE* expression was non-existent (i.e., equivalent to the negative control, parent *adhE* deletion strain, LL1111, Fig. 2(A)).

**Fig. 3:**
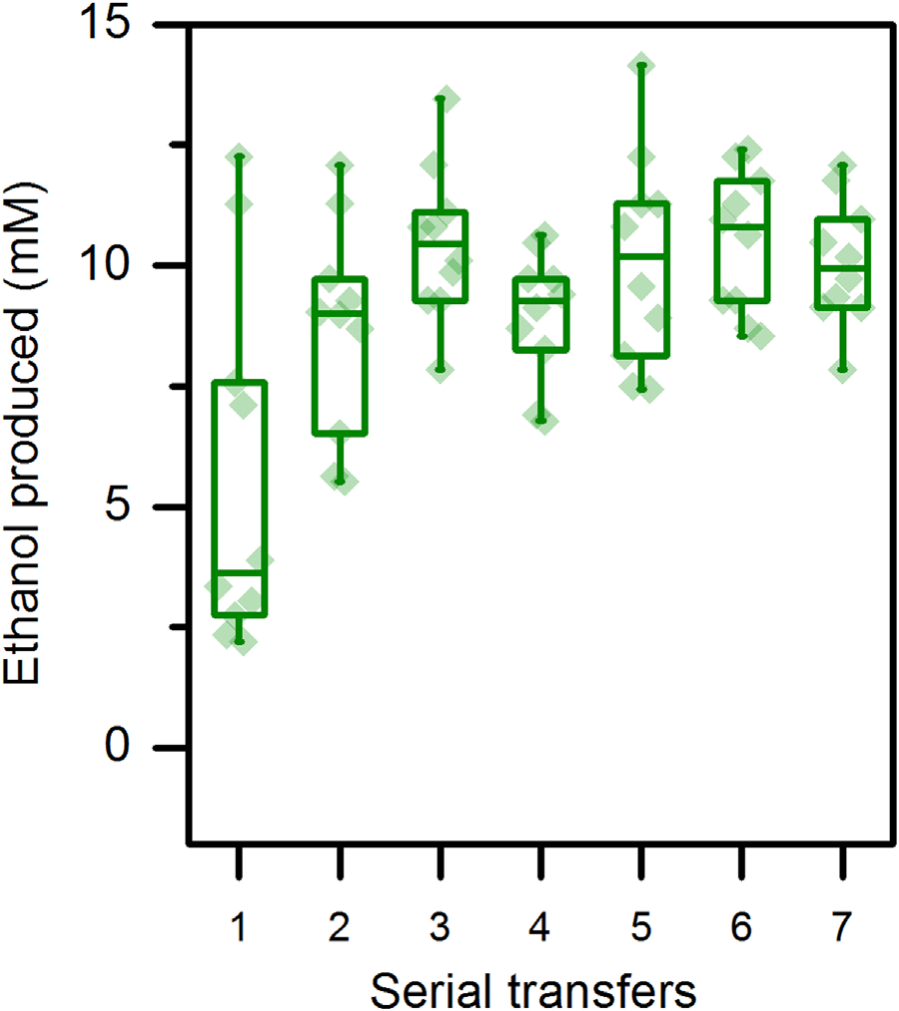
Ethanol production of strain LL1111 (*adhE* deletion) with plasmid pLL1119 (wild type Cth *adhE*) over several serial transfers. 10 colonies were subjected to daily serial transfers in CTFÜD medium with added thiamphenicol; each transfer was cultured for a total of 72 h before ethanol production was measured.

The effect of serial transfer is shown in Fig. 2(C). Although colonies showed a range of ethanol production levels upon initial transformation, several rounds of serial transfer caused ethanol production to converge on a single value (Fig. 3) that was similar to that of wild type. Regardless of the initial amount of ethanol production, after about 3 rounds of serial transfer, ethanol production had stabilized (Fig. 3). Differences in ethanol production were not due to differences in cellobiose consumption; in all cases where we measured cellobiose consumption, we found it was >95% complete.

### 3.4 Expressing different *adhEs* in strain LL1111

**Fig. 4:**
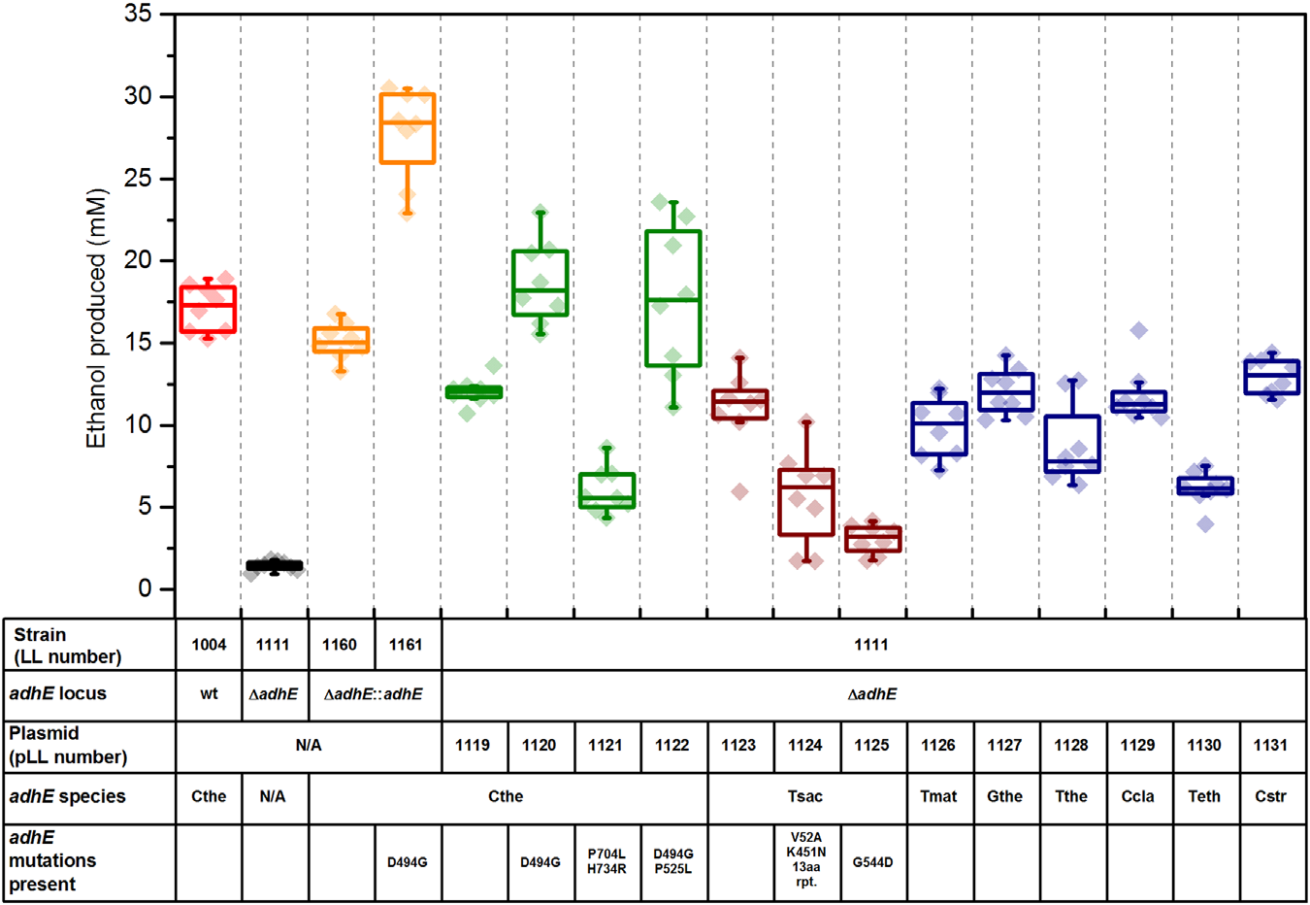
Ethanol production as a result of expressing an *adhE* gene in the *C. thermocellum adhE* deletion strain LL1111. Strains LL1160 and LL1161 show complementation of the *adhE* deletion with either wild type *adhE* or the D494G mutant *adhE*, and have been described previously (Lo et al., 2015, Zheng et al., 2015). For each condition, 8 colonies were assayed. Data for each colony is represented as a single point and was measured in biological triplicate experiments (error bars not shown on individual data points for clarity). For each experiment, ethanol was measured in duplicate assays. The box plot shows the 25–75th percentile range. Whiskers on the box plot represent 1.5× the interquartile range. *adhE* species are as follow: C the – *C. thermocellum*, Tsac – *T. saccharolyticum*, Tmat – *T. mathranii*, Gthe – *G. thermoglucosidasius*, The – *T. thermosaccharolyticum*, Ccla – *C. clariflavum*, Teth – *T. ethanolicus*, Cstr – *C. straminisolvens.*

With an improved expression plasmid, we tested whether ethanol production could be improved by using different *adhE*s; we chose 12 different *adhE*s (Table 1) and cloned them into plasmid pDGO144 under the control of the strong Clo1313_2638 promoter (Olson et al., 2015), and transformed these plasmids into the *adhE* deletion strain LL1111. We observed that the *C. thermocellum* D494G *adhE* gave the best ethanol production, consistent with previous reports (Zheng et al., 2015), which we attribute to an increase in NADPH-linked ADH activity. Another mutation, P525L, when combined with the D494G mutation, had the effect of increasing ethanol production in some colonies, but the overall effect was more varied (Fig. 4); this new *adhE* mutation (D494G P525L) came from the strain LL1231 (∆*hpt* ∆*hydG* ∆*ldh* ∆*pfl* ∆(*pta-ack*)), which was a strain evolved for high ethanol production by 2000 generations of serial transfer in 50 g/L cellobiose MTC-5 medium (unpublished data). With plasmid pLL1121 (*adhE* P740L H734R), despite being from an ethanol tolerant *C. thermocellum* strain (Brown et al., 2011), we nonetheless observed poorer performance compared to the other *C. thermocellum adhE*s, consistent with reported values; we suspect this is due to the decreased NADH-linked ADH activity of the mutant AdhE P740L H734R protein (Zheng et al., 2015).

The mutant *T. saccharolyticum adhE*s used in this study were both taken from strains that had been engineered for high ethanol yield (Shaw et al., 2012, Shaw et al., 2008b), it may therefore be surprising that we observed that these *adhE*s did not result in high ethanol production in strain LL1111. A recent report (Zheng et al., 2015) that characterized these two *adhE*s noted that not only had both *adhE*s undergone a change in cofactor preference, but also the overall NAD(P)H-linked ADH activity had decreased relative to wild type. One potential explanation for low ethanol production from the *T. saccharolyticum adhE* genes is that their NADPH-linked cofactor specificity is not compatible with the NADPH supply in *C. thermocellum*. Another possibility is that the reduced specific ADH activity results in decreased ethanol production (note that in *T. saccharolyticum*, this may be partly ameliorated by ethanol production from other ADH enzymes).

**Fig. 5:**
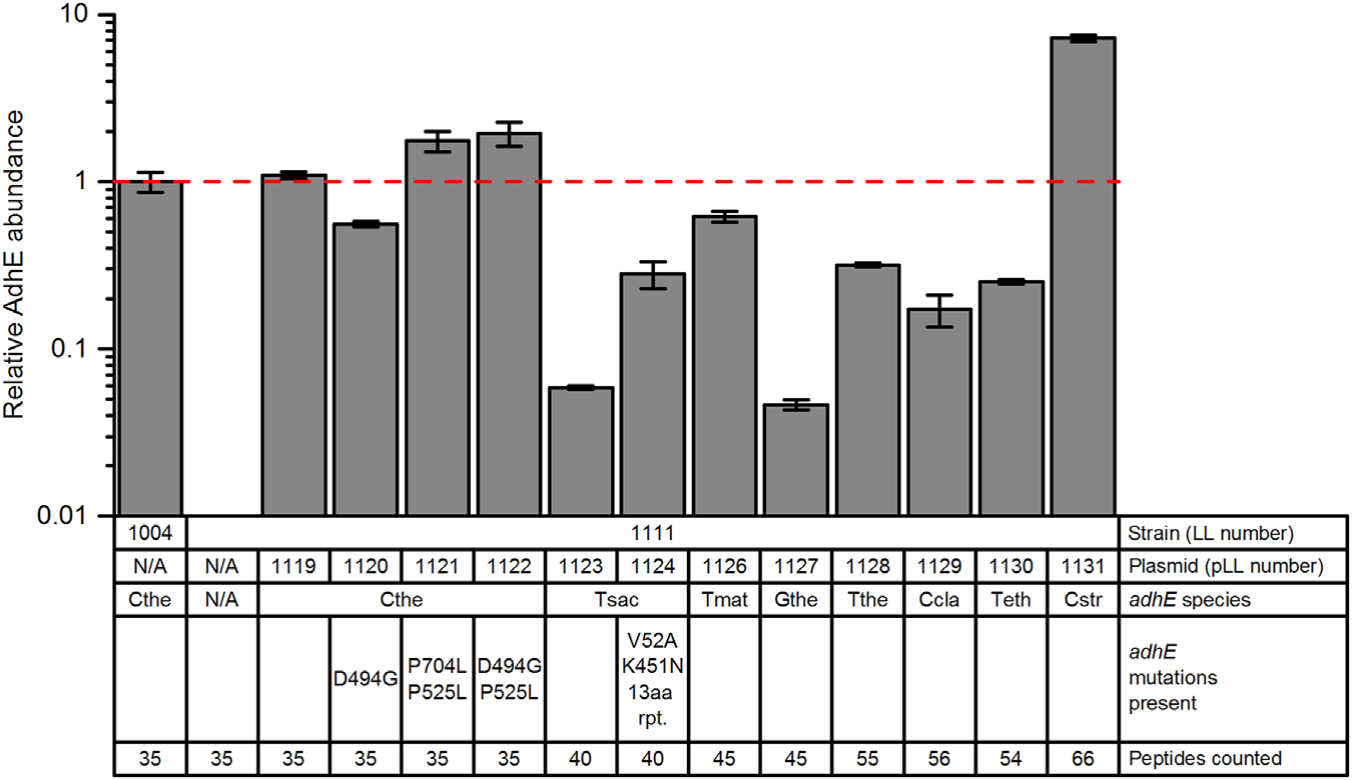
Relative abundances of AdhE peptides in representative samples of each strain normalized against wild type strain LL1004 levels. Values based on technical duplicate reads of one biological sample per strain; error bars depict standard deviation. Strain LL1111 (*adhE* deletion) with plasmid pLL1125 (*Tsac adhE* G544D) is not represented in this data set.

It is also possible that differences in AdhE protein levels in the various strains resulted in the differences in ethanol production. Abundance of each AdhE protein was measured by tandem mass spectrometry (Fig. 5, Table S2). In general, AdhE proteins originating from strains of *C. thermocellum* were expressed at high levels (equivalent to AdhE expression in wild-type *C. thermocellum*). Exogenous AdhE proteins were expressed at moderate levels (5–50% of wild-type *C. thermocellum* AdhE levels). Note that this still a very high level. Even the proteins expressed at the lowest level relative to *C. thermocellum* AdhE (i.e. AdhE from *T. saccharolyticum* from plasmid pLL1123 and from *G. thermoglucosidasius* from plasmid pLL1127) were still expressed in the top 30th percentile of protein expression in their respective strains (Table S2). Although there is clearly room for improvement in expression levels of several AdhEs, these results demonstrate the utility of our expression plasmid.

To determine if increases in ethanol production were related to changes in other fermentation products, we analyzed cultures of each strain by HPLC to measure liquid fermentation products. Ethanol, acetate, lactate and formate accounted for the majority of fermentation products. Even in the strains with the highest levels of ethanol production (LL1111 with plasmid pLL1120 (*adhE* D494G) and pLL1122 (*adhE* D494G P525L)), substantial lactate and acetate production remained (Table 3). It has been shown that lactate and acetate production in *C. thermocellum* can be eliminated by gene deletion (Argyros et al., 2011), and this may be an interesting direction for future work.

**Table 3:** Comparison of fermentation products. Cultures were grown on MTC medium with 14.12±0.98 mM initial cellobiose concentration for 72 h; no residual cellobiose was detected in any of the cultures i.e., cellobiose was fully consumed in all cases. Standard deviations calculated from sample size of 3. ND: fermentation product was not detected or below threshold of detection.

**Strain**	**Plasmid**	**Fermentation products (mM)**						
		**Ethanol**	**Acetate**	**Lactate**	**Formate**	**Pyruvate**	**Malate**	**Succinate**
LL1004	N/A	16.67±4.39	13.84±0.57	0.48±0.01	11.52±0.65	0.43±0.02	0.74±0.02	0.01±0.00
LL1111	N/A	0.52±0.00	9.95±0.11	30.17±0.31	1.10±0.02	0.32±0.00	0.49±0.13	0.10±0.00
LL1111	pLL1119	11.64±1.17	8.98±1.00	13.78±2.26	5.11±1.59	0.38±0.03	0.47±0.14	0.05±0.04
LL1111	pLL1120	17.07±5.36	10.43±3.89	7.86±1.38	9.16±4.01	0.56±0.12	0.43±0.07	0.07±0.00
LL1111	pLL1121	5.61±0.91	10.87±2.96	18.22±3.26	5.96±2.17	0.63±0.36	0.46±0.11	0.07±0.01
LL1111	pLL1122	21.18±5.89	5.59±1.06	12.02±2.28	2.86±0.77	0.40±0.02	0.39±0.07	0.06±0.01
LL1111	pLL1123	9.26±3.83	12.36±4.81	15.56±2.10	6.38±3.28	0.39±0.06	0.58±0.02	0.07±0.00
LL1111	pLL1124	7.22±1.89	7.89±1.35	22.30±2.15	2.13±0.68	0.40±0.01	0.36±0.07	0.07±0.00
LL1111	pLL1125	4.77±0.86	7.91±1.22	22.79±1.01	2.24±0.55	0.46±0.05	0.66±0.09	0.07±0.01
LL1111	pLL1126	9.66±2.88	8.09±1.48	18.94±0.23	2.62±0.62	0.44±0.08	0.36±0.08	0.02±0.04
LL1111	pLL1127	12.00±1.40	6.89±0.97	17.50±0.99	2.71±0.79	0.48±0.07	0.33±0.01	0.07±0.00
LL1111	pLL1128	9.47±1.61	10.48±1.69	16.00±3.48	4.59±2.11	0.42±0.04	0.57±0.12	0.06±0.00
LL1111	pLL1129	11.57±1.39	8.21±1.48	16.28±2.43	3.83±1.29	0.42±0.02	0.56±0.23	0.06±0.00
LL1111	pLL1130	7.26±0.80	8.25±0.63	21.28±1.09	2.42±0.34	0.44±0.02	0.52±0.13	0.04±0.04
LL1111	pLL1131	13.45±3.73	6.31±0.46	18.85±3.78	2.33±0.10	0.36±0.01	0.28±0.00	0.07±0.00

## 4 Conclusion

We successfully expressed a variety of *adhE* genes to evaluate their abilities to improve ethanol production in an *adhE* deletion strain of *C. thermocellum*. Although we did not find any *adhE*s that were substantially better than previous reports (Lo et al., 2015, Zheng et al., 2015), our ability to do this with a replicating plasmid will allow for faster progress in future metabolic engineering work.
