# Supplementary material for: Development of a plasmid-based expression system in Clostridium thermocellum and its use to screen heterologous expression of bifunctional alcohol dehydrogenases (adhEs)
Source: Metab Eng Commun. 2016 Apr 22;3:120–9. doi: 10.1016/j.meteno.2016.04.001 (PMC5678826; doi:10.1016/j.meteno.2016.04.001)
Supplement: Supplementary file 1 — Supplementary material [file mmc1.docx]

**Supplemental figures and tables**

**Figure S1.** Maps of the plasmids described in this work. Yellow arrows correspond to coding sequences, green arrows represent promoter sequences, and red arrows represent origins of replication. The “cloning site” annotation, indicated by blue boxes on the plasmid maps, denotes either a multiple cloning site, or a PvuII restriction site. In certain plasmid builds, the MCS or PvuII had disrupted the *cat* promoter, which we believe had contributed to these plasmids’ poor transformation efficiency (Table 2). Numbers above the plasmid indicate the coordinates in units of base pairs.


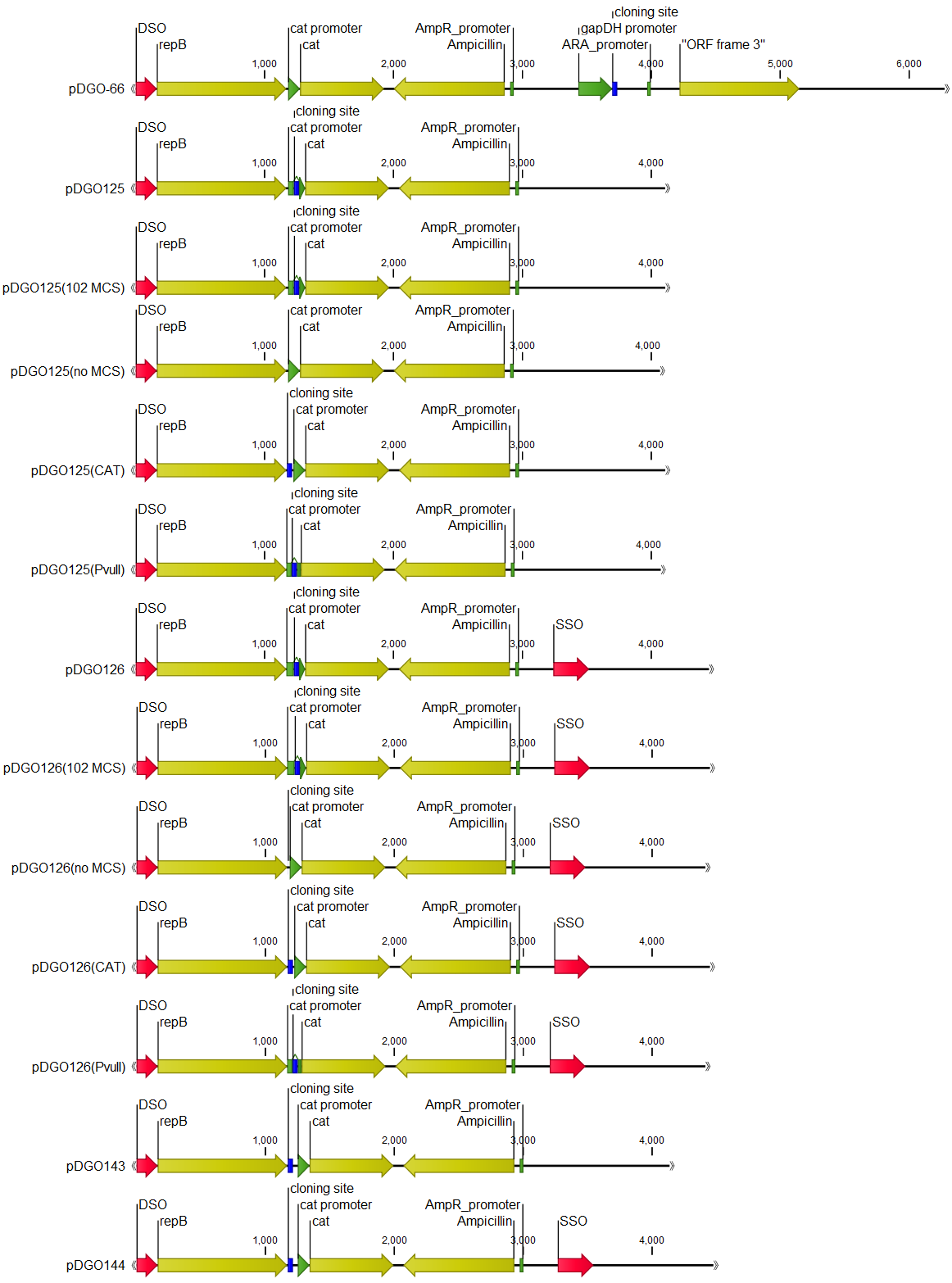


**Figure S2.** Relative protein abundances of glyceraldehye-3-phosphate dehydrogenase (GapDH) (S2A) and Phosphofructokinase (Pfk) (S2B); protein abundances are normalized against that of strain LL1004 (wild type control). Data shown is from technical duplicate measurements of one biological sample per strain. Error bars represent standard deviation. Strain LL1111 + plasmid pLL1125 is not represented in this data set.

**S2A.**

**
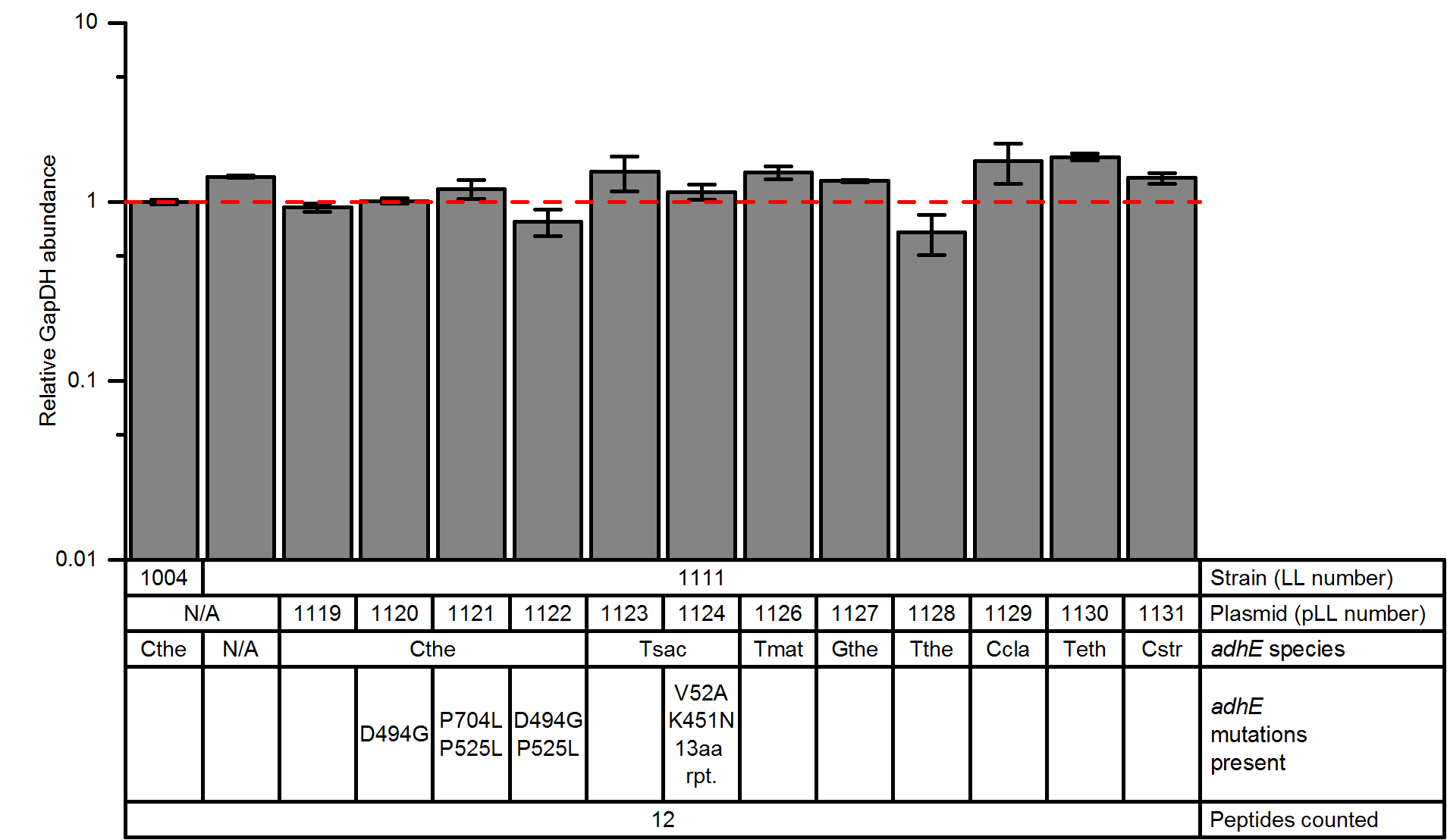
**

**S2B.**

**
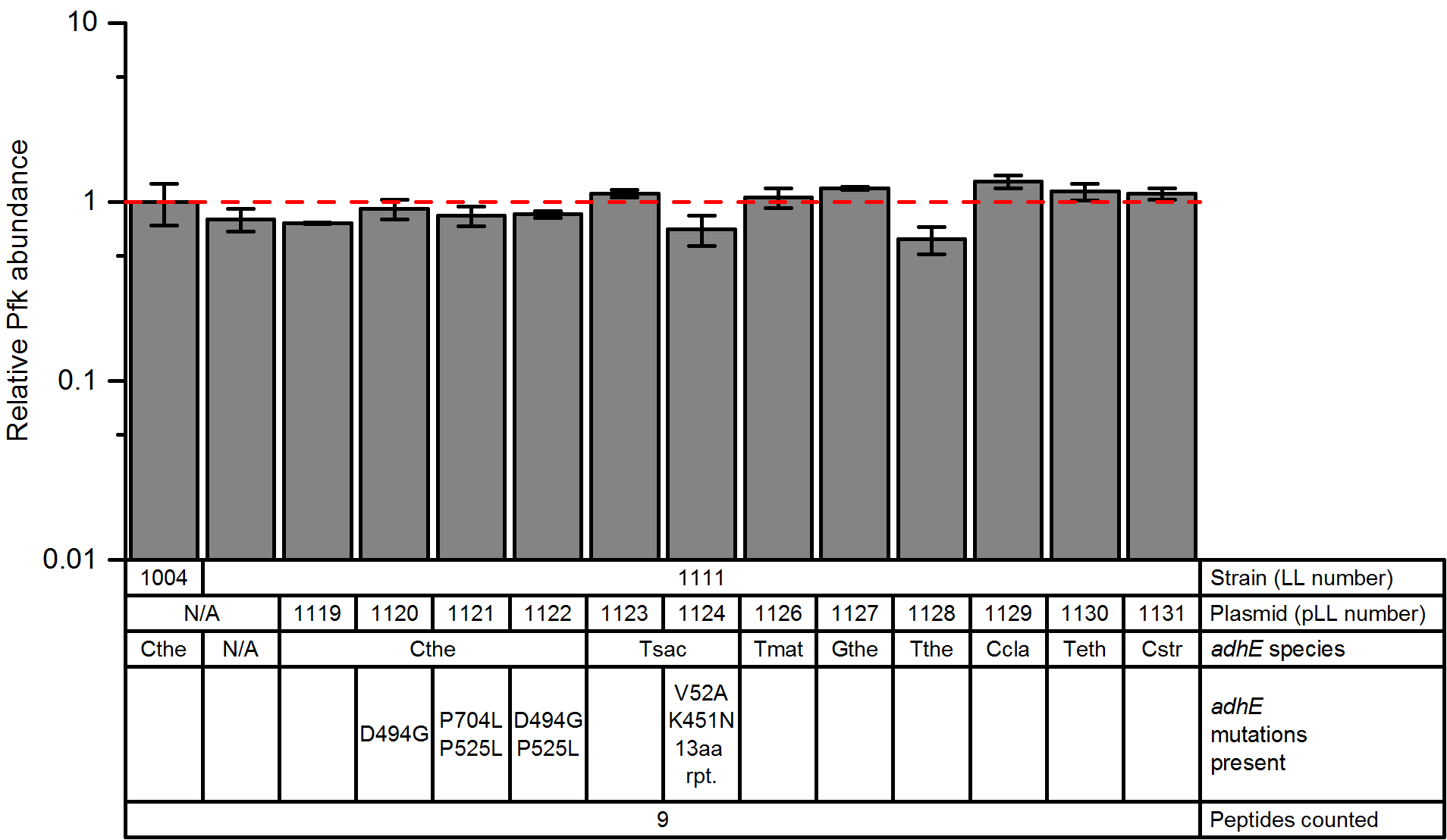
**

**Figure S3.** (S3A) Whole genome resequencing of strains LL1153 indicated that the strain was a mixed culture in which ~10% of the population carried an intact pSH007 plasmid, and the remainder possessing a truncated pSH007 plasmid. Figure S3B shows the plasmid maps of pSH007 both in its original form and in the truncated form; the deleted region of pSH007 is highlighted by the red arrow in the original plasmid map and described as “LL1153 adhE deletion region.”

**S3A.**


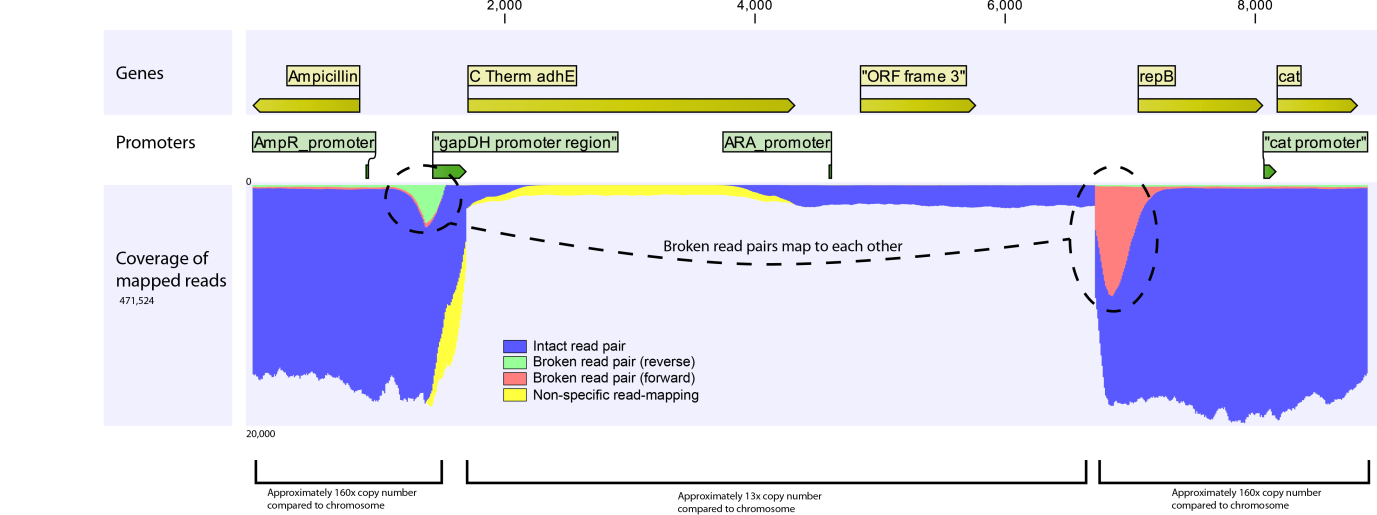


**S3B.**


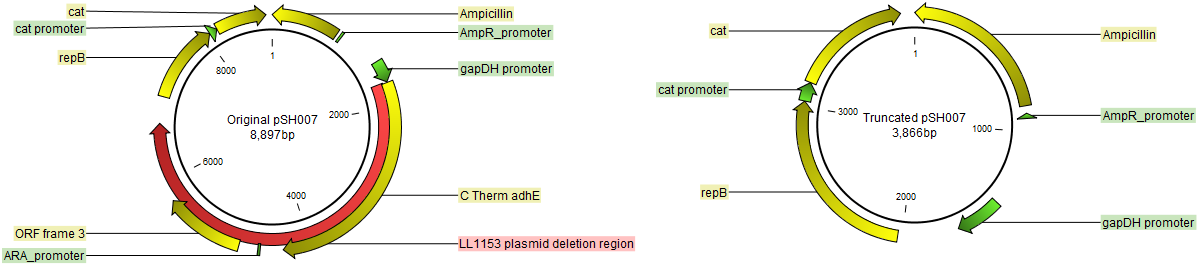


**Figure S4.** In strain LL1154, whole genome resequencing indicated that plasmid pSH007 had been integrated onto the chromosome at the *gapDH* promoter region, possibly through a single crossover event mediated by homology between the chromosomal locus and the plasmid-bound *gapDH* promoter.


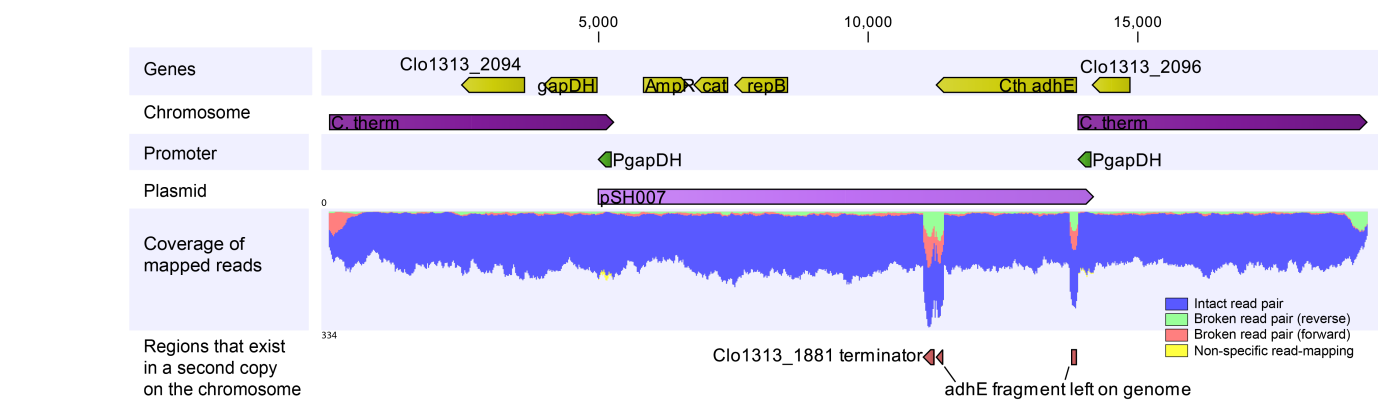


**Figure S5.** PCR analyses of plasmids pDGO143 and pDGO144 (here annotated with their alternate names, pDGO125’ins’ and pDGO126’ins’ respectively) as purified from *E. coli*, or from *C. thermocellum*. We were looking for any changes in PCR product size that would indicate an insertion or deletion mutation. The lack of difference between PCR product sizes obtained from the different template sources suggests that there are no major alterations to the plasmid sequences. The plasmid maps for pDGO143 and pDGO144 show the primer binding sites.


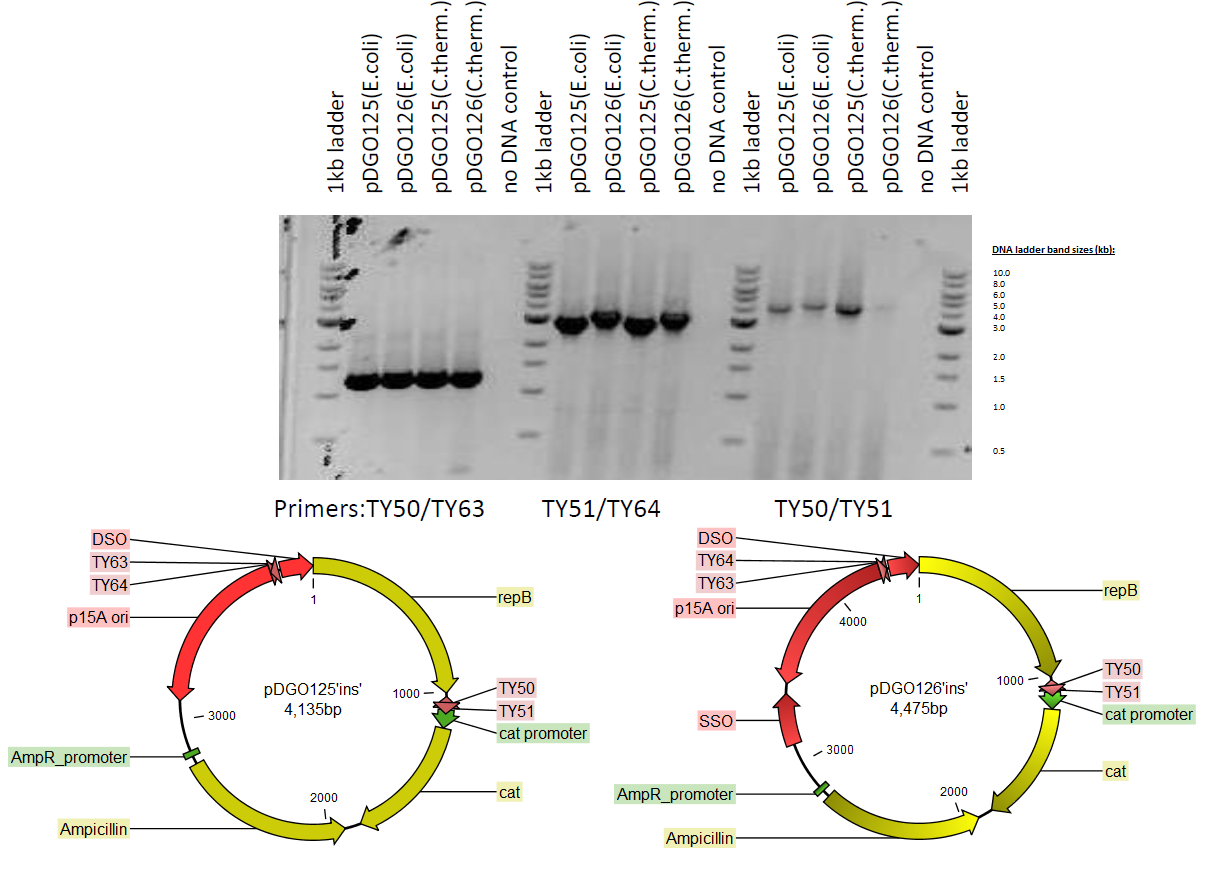


**Table S1.** List of primers used in this study.

| **Primers** | **Sequence (5'-3')** | **Description** |
| --- | --- | --- |
| XSH0039 | AGGCGATATTAATGCAGATGACGAAAATAGCGAATAAATAC | Forward primer to clone *C. thermocellum adhE* into pDGO-66 |
| XSH0040 | CCTGTTTATTTTAAACCAGTCATTTCTTCGCACCTCC | Reverse primer to clone *C. thermocellum adhE* into pDGO-66 |
| XSH0210 | GCCGAATGATGCAATATAAA | Forward primer to verify presence of plasmid, binds upstream of pDG0125/126 MCS |
| XSH0211 | CTTCTTGTTTTATGTTTCGGTA | Reverse primer to verify presence of plasmid, binds downstream of pDG0125/126 MCS |
| XSH0237 | AACAAATTCCTCCTTACTTTTG | clo1313_2638 promoter R |
| XSH0261 | GCAGGCATGCAAGCTGATAAACAAAGGACGGTTCAG | clo1313_2638 promoter F with 5' homology to pDGO144 HindIII site |
| XSH0238 | AAGGAGGAATTTGTTATGACGAAAATAGCGAATAAATAC | Forward primer to clone *C. thermocellum adhE* into pDGO144 |
| XSH0239 | AATTATTTCTATTAATCATTTCTTCGCACCTCC | Reverse primer to clone *C. thermocellum adhE* into pDGO144 |
| XSH0240 | AAGGAGGAATTTGTTATGGCAACGACAAAAACG | Forward primer to *clone T. saccharolyticum adhE* into pDGO144 |
| XSH0241 | AATTATTTCTATTAACTATGCACCGTATGCTTTTC | Reverse primer to clone *T. saccharolyticum adhE* into pDGO144 |
| XSH0242 | AAGGAGGAATTTGTTATGCCTACTTTATTACAAGAAAAAAAG | Forward primer to clone *T. mathranii adhE* into pDGO144 |
| XSH0243 | AATTATTTCTATTAATTATTCTCCATAGGCTTTTCTATATATTTC | Reverse primer to clone *T. mathranii adhE* into pDGO144 |
| XSH0244 | AAGGAGGAATTTGTTATGGCTGTGGAGGAAAG | Forward primer to clone *G. thermoglucosidasius adhE* into pDGO144 |
| XSH0245 | AATTATTTCTATTAATTAAACTCCTTTAAACGCTTG | Reverse primer to clone *G. thermoglucosidasius adhE* into pDGO144 |
| XSH0246 | AAGGAGGAATTTGTTATGGCAACGACAAAAATG | Forward primer to clone *T. thermosaccharolyticum adhE* into pDGO144 |
| XSH0247 | AATTATTTCTATTAATTATGCACCGTATGCTTTTC | Reverse primer to clone *T. thermosaccharolyticum adhE* into pDGO144 |
| XSH0248 | AAGGAGGAATTTGTTATGATTATGAAAGAAGCTATTAGG | Forward primer to clone *C. clariflavum adhE* into pDGO144 |
| XSH0249 | AATTATTTCTATTAATTATAAGCCATAAGCTTTAATG | Reverse primer to clone *C. clariflavum adhE* into pDGO144 |
| XSH0252 | AAGGAGGAATTTGTTATGCCTAACTTATTACAAGAAAG | Forward primer to clone *T. ethanolicus adhE* into pDGO144 |
| XSH0253 | AATTATTTCTATTAATTATTCTCCATAGGCTTTTC | Reverse primer to clone *T. ethanolicus adhE* into pDGO144 |
| XSH0254 | AAGGAGGAATTTGTTATGACAAATAGATACGAAGTTG | Forward primer to *clone C. straminisolvens adhE* into pDGO144 |
| XSH0255 | AATTATTTCTATTAATCATTCCTTCGCACCTC | Reverse primer to *clone C. straminisolvens adhE* into pDGO144 |
| TY50 | TAAAGTTAAACAAAATTATTTCTATTAAAGCTTGCATGCCTGCAGGTCGACTCTAGA | Primer #1 to screen plasmids pDGO143 and pDGO144 for rearrangements |
| TY51 | TAATAGAAATAATTTTGTTTAACTTTACAAACGGGATTGACTTTTAAAAAAGGATTG | Primer #2 to screen plasmids pDGO143 and pDGO144 for rearrangements |
| TY63 | ATGAGTGCTTTTTTTGCGTTTTGAGCGTAGCGAAAAACGA | Primer #3 to screen plasmids pDGO143 and pDGO144 for rearrangements |
| TY64 | TCGTTTTTCGCTACGCTCAAAACGCAAAAAAAGCACTCAT | Primer #4 to screen plasmids pDGO143 and pDGO144 for rearrangements |
| recA(new) F | TTTACGGCCAGGGTATTTCA | Forward primer for qPCR of *C. thermocellum recA* |
| recA(new) R | GCCAATCTTCTGACCGTTGT | Reverse primer for qPCR of *C. thermocellum recA* |
| *adhE* qPCR F | GCGTGGATATTTCCGAAGAA | Forward primer for qPCR of *C. thermocellum adhE* |
| *adhE* qPCR R | ATCCACCGTCAGCTACCAAC | Reverse primer for qPCR of *C. thermocellum adhE* |

**Table S2.** See external Excel spreadsheet “Supplementary Table S2”.
